# Functional replacement of myostatin with GDF-11 in the germline of mice

**DOI:** 10.1186/s13395-022-00290-z

**Published:** 2022-03-15

**Authors:** Se-Jin Lee, Adam Lehar, Renata Rydzik, Daniel W. Youngstrom, Shalender Bhasin, Yewei Liu, Emily L. Germain-Lee

**Affiliations:** 1 The Jackson Laboratory for Genomic Medicine, Farmington, CT USA; 2 Department of Genetics and Genome Sciences, University of Connecticut School of Medicine, Farmington, CT USA; 3 Department of Orthopaedic Surgery, University of Connecticut School of Medicine, Farmington, CT USA; 4 Brigham Research Assay Core Laboratory, Brigham and Women’s Hospital, Harvard Medical School, Boston, MA USA; 5 Research Program in Men’s Health: Aging and Metabolism, Boston Claude D. Pepper Older Americans Independence Center, Brigham and Women’s Hospital, Harvard Medical School, Boston, MA USA; 6 Department of Pediatrics, University of Connecticut School of Medicine, Farmington, CT USA; 7 Department of Reconstructive Sciences, Center for Regenerative Medicine and Skeletal Development, University of Connecticut School of Dental Medicine, Farmington, CT USA; 8 Division of Endocrinology & Diabetes and Center for Rare Bone Disorders, Connecticut Children’s, Farmington, CT USA

**Keywords:** Myostatin, GDF-11, Gene knock-in, Body composition, Muscle, Fat, Bone, Metabolism

## Abstract

**Background:**

Myostatin (MSTN) is a transforming growth factor-ß superfamily member that acts as a major regulator of skeletal muscle mass. GDF-11, which is highly related to MSTN, plays multiple roles during embryonic development, including regulating development of the axial skeleton, kidneys, nervous system, and pancreas. As MSTN and GDF-11 share a high degree of amino acid sequence identity, behave virtually identically in cell culture assays, and utilize similar regulatory and signaling components, a critical question is whether their distinct biological functions result from inherent differences in their abilities to interact with specific regulatory and signaling components or whether their distinct biological functions mainly reflect their differing temporal and spatial patterns of expression.

**Methods:**

We generated and characterized mice in which we precisely replaced in the germline the portion of the *Mstn* gene encoding the mature C-terminal peptide with the corresponding region of *Gdf11*.

**Results:**

In mice homozygous for the knock-in allele, all of the circulating MSTN protein was replaced with GDF-11, resulting in ~ 30–40-fold increased levels of circulating GDF-11. Male mice homozygous for the knock-in allele had slightly decreased muscle weights, slightly increased weight gain in response to a high-fat diet, slightly increased plasma cholesterol and HDL levels, and significantly decreased bone density and bone mass, whereas female mice were mostly unaffected.

**Conclusions:**

GDF-11 appears to be capable of nearly completely functionally replacing MSTN in the control of muscle mass. The developmental and physiological consequences of replacing MSTN with GDF-11 are strikingly limited.

## Background

Myostatin (MSTN) is a transforming growth factor-ß (TGF-ß) superfamily member that normally acts to limit skeletal muscle mass [1]. Mice lacking MSTN exhibit dramatic increases in muscle mass throughout the body, with individual muscles growing to about twice the normal size. *MSTN* has been highly conserved through evolution [2], and naturally occurring and engineered mutations in the *MSTN* gene also have been shown to result in increased muscle mass and/or function in numerous other mammalian [2–16], piscine [17–19], and avian [20, 21] species. Loss of MSTN leads to both an increase in the number of muscle fibers that are formed during development and an increase in the sizes of individual fibers [1]. Postnatally, MSTN is made by myofibers [1], circulates in the blood [22], and signals back to myofibers to limit growth [23, 24]. Based on this postnatal function of MSTN, numerous pharmaceutical and biotechnology companies have developed MSTN inhibitors that have been tested in clinical trials in patients with muscle and metabolic diseases (for review, see ref. [25]).

GDF-11, which was originally identified using *Mstn* as a probe [1], is a highly related TGF-ß family member that is about 90% identical to MSTN in the mature portion of the protein [26, 27]. Gene targeting studies in mice showed that the function of GDF-11 is distinct from that of MSTN. During embryogenesis, GDF-11 has been shown to regulate anterior-posterior patterning of the axial skeleton [28] as well as the development of the kidney [29], pancreas [30, 31], and nervous system [32–34]. Although the phenotypes of *Mstn*^*−/−*^ and *Gdf11*^*−/−*^ mice appear to be mostly non-overlapping, the two genes have been shown to be at least partially functionally redundant with respect to anterior-posterior axial patterning [35]. Much less is known about adult functions of GDF-11, as *Gdf11*^*−/−*^ mice die within the first 24 h after birth.

MSTN and GDF-11 are nearly indistinguishable in cell culture assays and also share many regulatory and signaling mechanisms and components. MSTN is synthesized in precursor form, and following proteolytic processing, the C-terminal dimer, which is the actual signaling molecule, remains non-covalently bound to the propeptide in an inactive, latent state [36, 37]. The MSTN latent complex can be activated by proteolytic cleavage of the propeptide by members of the BMP-1/tolloid family of metalloproteases [38], which appears to be the dominant mechanism operating in vivo [39]. Similarly, the C-terminal dimer and propeptide of GDF-11 also form a latent complex that can be activated by BMP-1/tolloid proteases [40]. MSTN is regulated extracellularly by other binding proteins as well, including follistatin (FST) [36], FSTL-3 [41], GASP-1, and GASP-2 [42]. Genetic studies have shown that loss of FST [43–45] and/or GASP-1/GASP-2 [46] results in decreases in muscle mass and fiber type shifts consistent with their roles in inhibiting MSTN in vivo. These binding proteins are also capable of inhibiting GDF-11, and mice lacking FST [43] or GASP-1/GASP-2 [46] also exhibit axial patterning defects consistent with increased activity of GDF-11. Finally, when free of inhibitory binding proteins, MSTN is capable of binding the type 2 receptors, ACVR2 and ACVR2B [36], and the type 1 receptors, ALK4 and ALK5 [47]. Targeting these receptors in mice leads to increased muscle mass, consistent with the key roles that these receptors play in mediating MSTN signaling in vivo [24, 48]. GDF-11 is capable of binding to these same receptors, and *Acvr2* and *Acvr2b* mutant mice exhibit axial patterning and kidney defects [49–53] similar to those seen in *Gdf11* mutants.

Given that MSTN and GDF-11 share a high degree of amino acid sequence identity, behave virtually identically in cell culture assays, and utilize similar regulatory and signaling components, a critical question is whether their distinct biological functions result from inherent differences in their abilities to interact with specific regulatory and signaling components or whether their distinct biological functions mainly reflect their differing temporal and spatial patterns of expression. Given that both MSTN and GDF-11 circulate in the blood, another critical question is whether these ligands act locally or systemically in signaling to target cells. Genetic studies have suggested that MSTN has both autocrine/paracrine and endocrine modes of action [54], although very little is known about the functions of GDF-11 and its mode of action in adult animals. Although overexpression of either MSTN [22] or GDF-11 [55, 56] has been shown to induce a cachexia-like syndrome in adult mice, at least one study has reported distinct effects of giving MSTN and GDF-11 protein exogenously to mice, suggesting that these two molecules may have inherent differences in their biological properties [57]. Here, we addressed the functional equivalence of these two molecules by replacing the portion of the *Mstn* gene encoding its mature domain with the corresponding portion of *Gdf11* in the germline of mice.

## Methods

All animal experiments were carried out in accordance with protocols that were approved by the Institutional Animal Care and Use Committees at the University of Connecticut School of Medicine and Johns Hopkins University School of Medicine. To generate mice carrying a *Mstn*^*Gdf11*^ knock-in allele, the targeting construct was electroporated into embryonic stem (ES) cells, and ES cell colonies carrying the homologously targeted allele were injected into blastocysts. Chimeric mice generated from these blastocysts were bred to identify those exhibiting germline transmission of the targeted allele. Offspring from these matings were then bred with *EIIa-Cre* transgenic mice [58] in order to delete the neomycin resistance cassette in the germline. From these crosses, we obtained mice carrying the *Mstn*^*Gdf11*^ knock-in allele lacking the neo-cassette.

Circulating MSTN and GDF-11 levels were determined using a liquid chromatography-tandem mass spectrometry assay, as described [59]. There was no detectable cross-reactivity of MSTN in the GDF-11 assay or of GDF-11 in the MSTN assay; the addition of up to 100 ng/mL of GDF-11 did not significantly affect GDF-8 measurement, and addition of up to 100 ng/mL GDF-8 did not affect GDF-11 measurement. The lower limit of quantitation was 0.5 ng/mL for GDF-8 as well as for GDF-11. The linear range of the assay was from 0 to 100 ng/mL for both. The inter-assay coefficients of variation in the MSTN assay were 15.1%, 11.3%, and 7.4% in the low- (8.7 ng/mL), medium- (51.1 ng/mL), and high- (97.6 ng/mL) quality control pools, respectively, and the corresponding CVs in the GDF-11 assay were 8.7%, 12.8%, and 17.1% in the low- (3.4 ng/mL), medium- (52.0 ng/ml), and high- (104.7 ng/ml) quality control pools, respectively [59].

For measurement of muscle weights, individual muscles were dissected from both sides of 10-week-old mice, and the average weight was used for each muscle. Serial sections (15 μm) were cut transversely through the widest point of the gastrocnemius muscle using a cryostat. Fiber type analysis was carried out using antibodies (BA-D5, SC-71, and BF-F3 for myosin heavy chains type I, IIa, and IIb, respectively) developed by Schiaffino et al. [60] and obtained from the Developmental Studies Hybridoma Bank developed under the auspices of the National Institute of Child Health and Human Development and maintained by the University of Iowa. Live animal imaging was performed using a Piximus dual-energy X-ray absorptiometer (DXA). Glucose tolerance tests (GTT) were performed by administering an intraperitoneal injection of 1 g glucose/kg body weight following a 6-h fast. Mice were then placed on a 60-kcal % fat diet (D12492, Research Diets, Inc.), and a repeat GTT was performed after 4 weeks; mice were then maintained on the high-fat diet for an additional 4 weeks. For analysis of skeletal patterning, newborn mice were euthanized, skinned, eviscerated, fixed in 80% ethanol, and dehydrated in 95% ethanol for 1 day and acetone for 3 days. Skeletons were stained in 10% acetic acid in ethanol containing 0.003% Alizarin red and 0.0045% Alcian blue for 36 h. After staining, skeletons were cleared in 1% potassium hydroxide and transferred to 20%, 50%, 80%, and 100% glycerol over several days. For microCT analysis, the left femur, left humerus, and lumbar vertebrae were placed in 70% ethanol. μCT was performed in a Scanco μCT40 at 8 μm^3^ resolution. Samples were scanned in 70% ethanol 55 kVp, 145 μA intensity, and 300 ms. The instrument is calibrated weekly using Scanco phantoms, and all scans passed routine quality control verification. Analysis of bones was conducted using standard protocols, with a lower threshold of 2485 HU for femoral trabeculae, 4932 HU for femoral cortex, and 3078 HU for vertebral trabeculae [61]. Surface renderings were generated corresponding to each of these thresholds. For all data, statistical significance was assessed using Student’s *t* test.

## Results, discussion, and conclusions

**Fig. 1:**
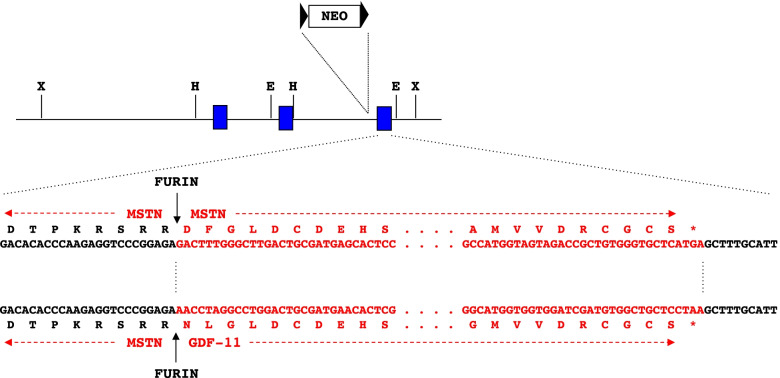
Schematic of the *Mstn* locus with the locations of the coding segments shown as blue boxes and the sequence of the coding portion of exon 3 shown below. The segment encoding the mature C-terminal domain following the furin processing site is shown in red. In the targeting construct, this segment was replaced precisely with the corresponding segment taken from *Gdf11*. The neo-cassette, introduced into intron 2 for selection of ES cells, was removed using a germline-expressed cre recombinase, leaving behind a single LoxP site in intron 2. All studies were carried out after crossing the neo-deleted allele (*Mstn*^*Gdf11*^) onto a C57BL/6 genetic background. X, XbaI; H, HindIII; E, EcoRI

As one approach to determine whether there are fundamental inherent differences between MSTN and GDF-11 that can lead to distinct biological activities in vivo, we analyzed the effect of replacing MSTN with GDF-11 in the germline. As shown in Fig. 1, the *Mstn* gene contains three exons, with the mature C-terminal domain being encoded within exon 3. We generated a targeting construct in which we precisely replaced the coding sequence for the MSTN C-terminal domain, starting at the furin proteolytic processing site, with that encoding the GDF-11 C-terminal domain. Hence, this knock-in allele (*Mstn*^*Gdf11*^) encodes a hybrid precursor protein consisting of the MSTN propeptide and the GDF-11 C-terminal domain, and following proteolytic processing, the mature GDF-11 signaling molecule would be produced wherever MSTN would normally be produced. A previous study showed that the MSTN propeptide is capable of binding and inhibiting the GDF-11 C-terminal dimer [37], so our expectation was that the MSTN propeptide from this hybrid protein would be capable of maintaining the mature GDF-11 C-terminal dimer in a latent state, as is the case for mature MSTN [36]. Following homologous recombination in embryonic stem cells, injection of the targeted cells into blastocysts, and transfer of the blastocysts to pseudopregnant females, we obtained chimeric mice that transferred the knock-in allele (*Mstn*^*Gdf11*^) through the germline. After removing the neo-cassette using a germline expressed cre transgene [58], we backcrossed the knock-in allele 5 generations onto a C57BL/6 genetic background prior to analysis.

**Fig. 2:**
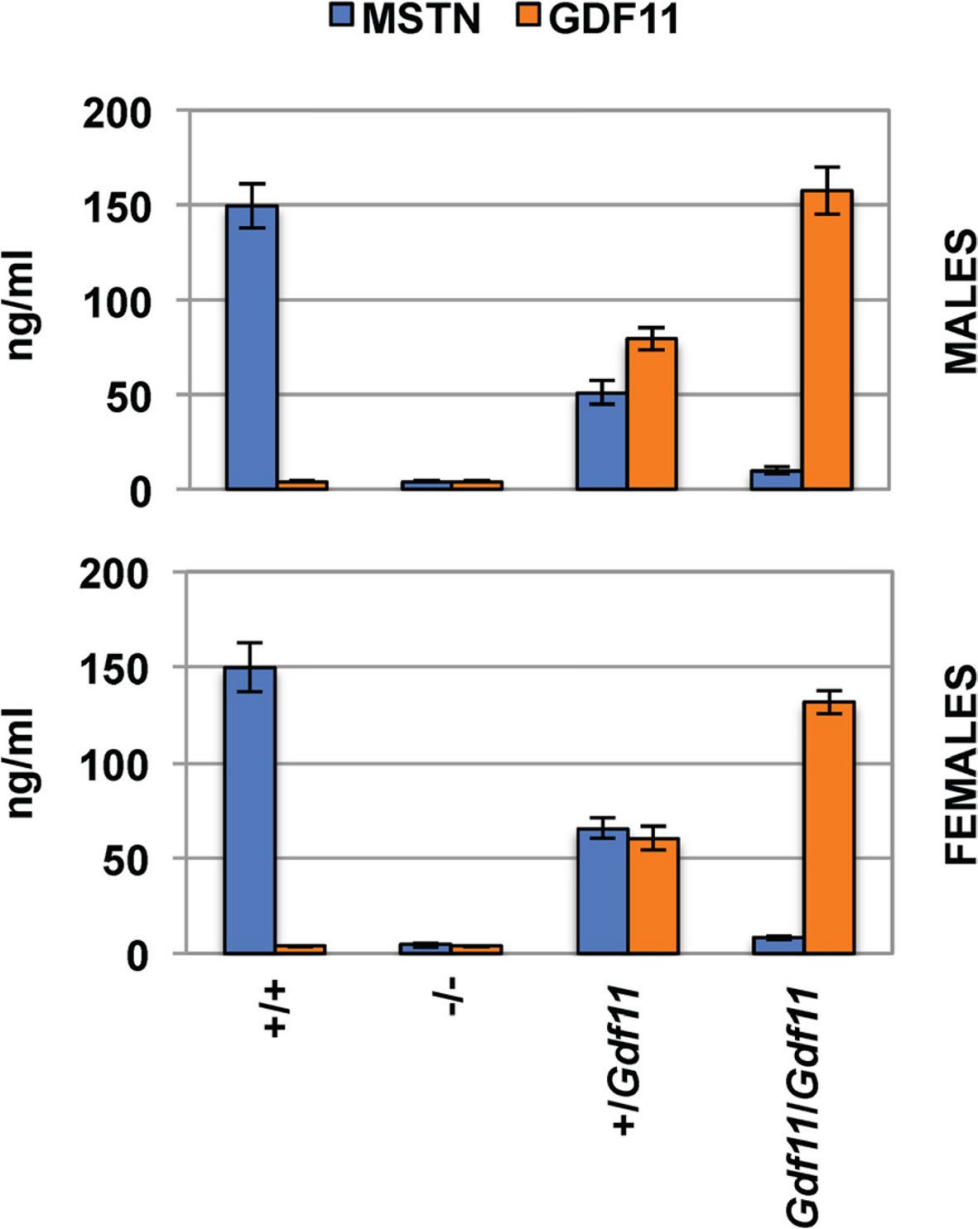
MSTN and GDF-11 plasma levels. Numbers of male (M) and female (F) mice were *n* = 6M, 6F for *Mstn*^*+/+*^, *n* = 6M, 6F for *Mstn*^*−/−*^, *n* = 6M, 4F for *Mstn*^*+/Gdf11*^, and *n* = 5M, 5F for *Mstn*^*Gdf11/Gdf11*^. *p* < 0.001 for all comparisons of MSTN or GDF-11 levels between groups, except for MSTN values between *Mstn*^*−/−*^ and *Mstn*^*Gdf11/Gdf11*^ mice and GDF-11 values between *Mstn*^*+/+*^ and *Mstn*^*−/−*^ mice, which were not significant

*Mstn*^*Gdf11/Gdf11*^ mice were viable, and we carried out all analysis on 10-week-old *Mstn*^*+/+*^, *Mstn*^*+/Gdf11*^, and *Mstn*^*Gdf11/Gdf11*^ mice that we generated from inter-crosses of *Mstn*^*+/Gdf11*^ mice. We first measured MSTN and GDF-11 protein levels in the plasma of these mice using a liquid chromatography-tandem mass spectrometry assay highly specific for each protein [59]. As shown in Fig. 2, wild-type mice had circulating MSTN and GDF-11 levels in the range of ~ 150 ng/ml and ~ 4 ng/ml, respectively. Mice carrying the *Mstn*^*Gdf11*^ allele showed circulating levels of MSTN and GDF-11 consistent with a complete replacement of MSTN with GDF-11. In particular, *Mstn*^*Gdf11/Gdf11*^ mice had no detectable circulating MSTN and had levels of circulating GDF-11 comparable to the normal levels of MSTN, which is ~ 30–40-fold higher than the normal circulating levels of GDF-11. *Mstn*^*+/Gdf11*^ mice had intermediate levels of both proteins.

**Fig. 3:**
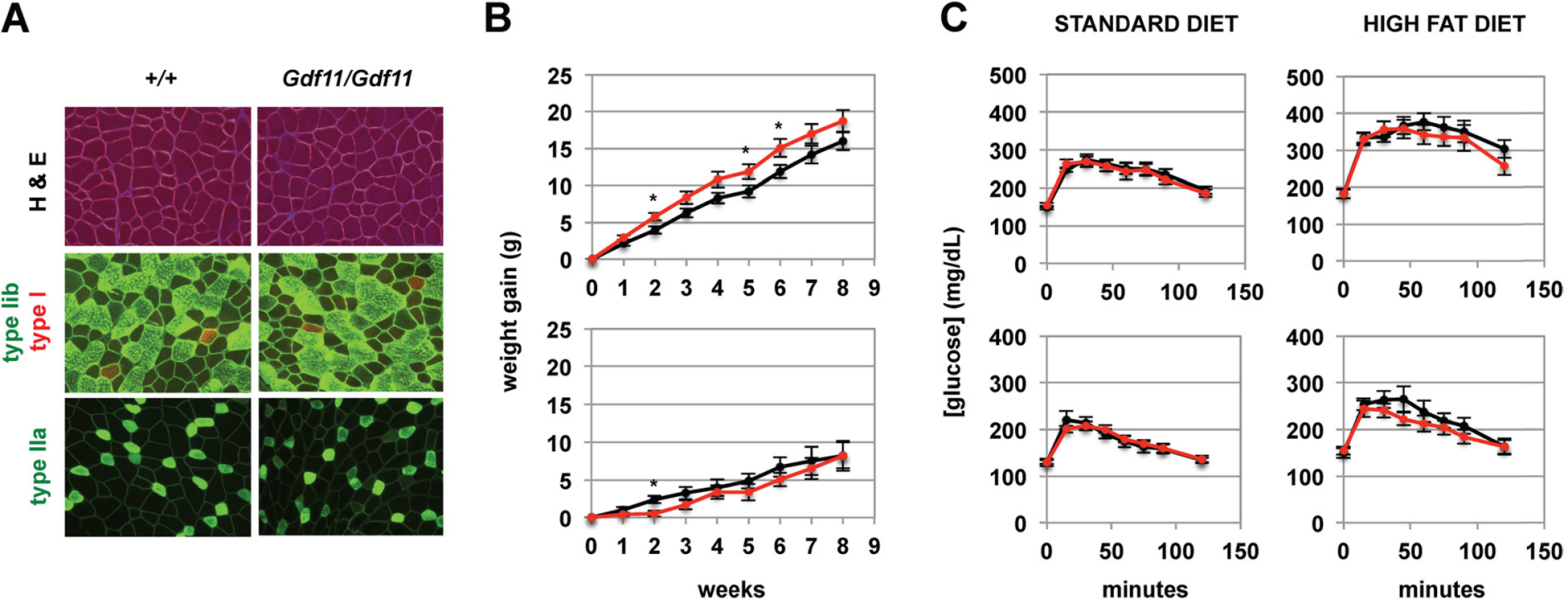
**A** Representative sections of gastrocnemius muscles stained with H&E (upper panels) or analyzed by immunofluorescence for different fiber types (middle and lower panels). Please note that the three images for a given genotype were not taken from adjacent sections. **B** Weight gain on high-fat diets for *Mstn*^*+/+*^ (*n* = 6M, 6F, black lines) and *Mstn*^*Gdf11/Gdf11*^ (*n* = 5M, 5F, red lines) mice. *p < 0.05. **C** Glucose tolerance tests on *Mstn*^*+/+*^ (black lines) and *Mstn*^*Gdf11/Gdf11*^ (red lines) mice. Numbers of mice were *n* = 9M, 6F for *Mstn*^*+/+*^, and *n* = 9M, 7F for *Mstn*^*Gdf11/Gdf11*^ for mice on standard diets, and *n* = 10M, 7F for *Mstn*^*+/+*^ and *n* = 9M, 7F for *Mstn*^*Gdf11/Gdf11*^ for mice on high-fat diets. None of the differences at any time point were statistically significant

To determine whether GDF-11 can functionally replace MSTN, we first examined body composition. In 10-week-old mice, total body weights and lean body mass (by DXA analysis) showed a small (~ 6–8%) but statistically significant decrease in *Mstn*^*Gdf11/Gdf11*^ compared to *Mstn*^*+/+*^ mice in males but not in females (Table 1). Consistent with these decreases in lean body mass, the *Mstn*^*Gdf11*^ allele had a dose-dependent effect on muscle mass in males, with individual muscles of *Mstn*^*Gdf11/Gdf11*^ mice weighing approximately 10% less than those of *Mstn*^*+/+*^ mice and *Mstn*^*+/Gdf11*^ mice exhibiting an intermediate effect (Table 2); differences in muscle weights between *Mstn*^*Gdf11/*Gdf11^ and *Mstn*^*+/+*^ female mice were not statistically significant. The fact that muscle weights were not increased in *Mstn*^*Gdf11/Gdf11*^ mice implies that GDF-11 can functionally substitute for MSTN in negatively regulating muscle mass. Moreover, the fact that weights are slightly decreased suggests that GDF-11 produced from this knock-in allele may even be slightly more active than endogenous MSTN. We also carried out fiber type analysis, as loss of *Mstn* has been shown to lead to shifts toward more glycolytic 2B fibers [62]. As shown in Fig. 3A and Table 3, we found no differences in the distribution of fiber types between *Mstn*^*Gdf11/*Gdf11^ and *Mstn*^*+/+*^ mice.

**Table 1:** DXA and fasting blood/plasma analysis

	Male +/*+*	Male *Gdf11*/*Gdf11*	Female +/*+*	Female *Gdf11*/*Gdf11*
Body weight (g)	25.2 ± 0.5 (23)	23.7 ± 0.3^b^ (30)	20.0 ± 0.3 (19)	19.5 ± 0.3 (26)
DXA				
Lean mass (g)	19.6 ± 0.6 (12)	18.0 ± 0.4^a^ (11)	15.7 ± 0.3 (8)	15.0 ± 0.3 (12)
Fat mass (g)	2.9 ± 0.2 (12)	3.0 ± 0.1 (11)	2.3 ± 0.1 (8)	2.3 ± 0.1 (12)
BMD (g/cm^2^)				
Whole body	0.0465 ± 0.0008 (12)	0.0443 ± 0.0005^a^ (11)	0.0447 ± 0.0003 (8)	0.0440 ± 0.0004 (12)
Right humerus	0.0405 ± 0.0008 (12)	0.0388 ± 0.0004 (11)	0.0395 ± 0.0006 (8)	0.0390 ± 0.0006 (12)
Left humerus	0.0444 ± 0.0011 (12)	0.0409 ± 0.0007^b^ (11)	0.0414 ± 0.0009 (8)	0.0419 ± 0.0007 (12)
Right femur	0.0693 ± 0.0025 (12)	0.0629 ± 0.0011^a^ (11)	0.0615 ± 0.0008 (8)	0.0608 ± 0.0006 (12)
Left femur	0.0756 ± 0.0026 (12)	0.0678 ± 0.0016^a^ (11)	0.0635 ± 0.0013 (8)	0.0627 ± 0.0006 (12)
L2 + L3 vertebrae	0.0537 ± 0.0011 (12)	0.0504 ± 0.0012^a^ (11)	0.0534 ± 0.0012 (8)	0.0534 ± 0.0011 (12)
L4 + L5 vertebrae	0.0580 ± 0.0010 (12)	0.0547 ± 0.0015 (11)	0.0602 ± 0.0023 (8)	0.0588 ± 0.0013 (12)
Glucose (mg/dL)				
Standard diet	160.9 ± 5.8 (18)	161.2 ± 3.3 (22)	127.8 ± 4.2 (13)	139.6 ± 4.1^a^ (17)
High-fat diet	207.6 ± 8.1 (10)	209.1 ± 6.1 (9)	148.3 ± 11.0 (7)	161.3 ± 10.9 (7)
Insulin (ng/ml)				
Standard diet	0.97 ± 0.17 (10)	0.93 ± 0.09 (14)	0.46 ± 0.18 (7)	0.25 ± 0.07 (10)
High-fat diet	3.33 ± 0.50 (10)	4.38 ± 0.63 (14)	0.61 ± 0.17 (7)	0.98 ± 0.35 (10)
Leptin (ng/ml)				
Standard diet	2.76 ± 0.66 (10)	4.04 ± 0.50 (14)	2.30 ± 0.49 (7)	2.60 ± 0.32 (10)
High-fat diet	52.82 ± 7.76 (10)	58.26 ± 9.47 (14)	20.93 ± 8.55 (7)	26.91 ± 8.58 (10)
Cholesterol (mg/dL)				
Standard diet	141.1 ± 3.5 (10)	168.6 ± 3.3^c^ (14)	117.7 ± 7.5 (7)	118.6 ± 5.3 (10)
High-fat diet	192.1 ± 11.5 (10)	237.4 ± 13.9^a^ (14)	139.3 ± 10.3 (7)	154.4 ± 9.6 (10)
HDL (mg/dL)				
Standard diet	98.9 ± 2.2 (10)	116.6 ± 1.4^c^ (14)	79.0 ± 4.5 (7)	78.8 ± 3.1 (10)
High-fat diet	131.7 ± 6.2 (10)	151.5 ± 5.5^a^ (14)	100.1 ± 6.4 (7)	109.1 ± 6.1 (10)
LDL (mg/dL)				
Standard diet	1.94 ± 0.46 (10)	2.03 ± 0.20 (14)	6.83 ± 0.74 (7)	7.08 ± 0.46 (10)
High-fat diet	2.90 ± 0.67 (10)	5.16 ± 0.82 (14)	5.32 ± 0.34 (7)	6.46 ± 0.62 (10)
Trigylcerides (mg/dL)				
Standard diet	76.6 ± 3.5 (10)	69.2 ± 1.8 (14)	69.4 ± 3.7 (7)	60.9 ± 3.8 (10)
High-fat diet	93.9 ± 3.0 (10)	87.6 ± 1.9 (14)	71.4 ± 5.3 (7)	71.3 ± 6.4 (10)
Free fatty acids (mEq/L)				
Standard diet	0.69 ± 0.06 (10)	0.61 ± 0.03 (14)	0.61 ± 0.06 (7)	0.71 ± 0.17 (10)
High fat diet	0.63 ± 0.02 (10)	0.57 ± 0.02 (14)	0.56 ± 0.05 (7)	0.55 ± 0.07 (10)

Numbers of mice analyzed are shown in parentheses

^a^*p* < 0.05 vs. +/+

^b^*p* < 0.01 vs. *+*/+

^c^*p* < 0.001 vs. +/+

**Table 2:** Muscle weights (mg)

	Pectoralis	Triceps	Quadriceps	Gastrocnemius
Males				
+/*+* (*n* = 11)	78.3 ± 2.1	108.3 ± 3.5	198.5 ± 5.9	138.4 ± 3.0
+/*Gdf11* (*n* = 28)	70.5 ± 1.1^b^	99.8 ± 1.7^a^	185.5 ± 3.0	130.9 ± 1.8^a^
*Gdf11*/*Gdf11* (*n* = 19)	65.4 ± 1.7^c^	97.6 ± 2.1^a^	173.7 ± 3.3^b^	121.5 ± 1.7^c^
Females				
+/*+* (*n* = 11)	52.9 ± 1.1	77.9 ± 1.0	148.8 ± 2.2	102.5 ± 1.4
+/*Gdf11* (*n* = 19)	51.8 ± 1.1	77.7 ± 1.5	146.8 ± 2.1	102.9 ± 1.8
*Gdf11*/*Gdf11* (*n* = 14)	49.5 ± 1.5	75.1 ± 1.2	141.6 ± 3.5	98.3 ± 2.3

^a^*p* < 0.05 vs. +/+

^b^*p* < 0.01 vs. *+*/+

^c^*p* < 0.001 vs. +/+

**Table 3:** Fiber type numbers in gastrocnemius muscles

	*+/+* (*n* = 3)	*Gdf11*/*Gdf11* (*n* = 3)
Total fibers	8295 ± 357	8136 ± 103
Type I fibers	130 ± 12	126 ± 7
Type IIa fibers	1446 ± 57	1540 ± 173
Type IIb fibers	6398 ± 431	6473 ± 405

None of the differences between *+/+* and *Gdf11*/*Gdf11* mice are statistically significant

In addition to regulating muscle mass, MSTN also regulates body composition in terms of fat mass, with *Mstn*^*−/−*^ mice [63] as well as mice in which MSTN receptors (ACVR2 and ACVR2B) have been targeted in myofibers [24] having reductions in total body fat. By DXA analysis, we observed no differences in total body fat content in *Mstn*^*Gdf11/Gdf11*^ compared to *Mstn*^*+/+*^ mice (Table 1). In addition, we found no significant differences in plasma leptin levels in the knock-in mice compared to *wild type* mice. *Mstn*^*−/−*^ mice have also been shown to exhibit less weight gain when placed on high-fat diets [64], which has also been seen in mice in which *Acvr2* and *Acvr2b* have been targeted in myofibers [24]. As shown in Fig. 3B, male *Mstn*^*Gdf11/Gdf11*^ mice actually gained slightly more weight than *Mstn*^*+/+*^ mice when placed on high-fat diets, although leptin levels were similar between *Mstn*^*Gdf11/Gdf11*^ and *Mstn*^*+/+*^ mice even after 8 weeks on the high-fat diet (Table 1); no significant differences in weight gains or leptin levels were seen in female mice placed on high-fat diets. We also observed small, but significant increases in plasma cholesterol and HDL levels in male *Mstn*^*Gdf11/Gdf11*^ mice maintained on standard diets compared to *Mstn*^*+/+*^ mice (increased by 19% and 18%, respectively) as well as following 8 weeks on a high-fat diet (increased by 24% and 15%, respectively) (Table 1). No differences were seen in LDL, triglyceride, or free fatty acid levels in male *Mstn*^*Gdf11/Gdf11*^ mice or in any of the lipid levels in female *Mstn*^*Gdf11/Gdf11*^ mice. Hence, although some small differences in both skeletal muscle mass and high-fat weight gain were seen in male mutant mice, replacement of the MSTN mature domain with that of GDF-11 had only a minor effect on overall body composition.

The MSTN-GDF-11 regulatory system is also known to have effects on glucose metabolism. In particular, *Mstn*^*−/−*^ mice are able to maintain normal or lower blood glucose levels despite having lower insulin levels [24, 63]. In addition, GDF-11 is known to play an important role in pancreatic development [30, 31], and administration of GDF-11, but not MSTN, protein to mice has been shown to improve glucose tolerance [57]. As shown in Table 1, fasting blood glucose levels were slightly higher in *Mstn*^*Gdf11/Gdf11*^ females compared to *Mstn*^*+/+*^ controls maintained on standard diets, but no statistically significant differences were seen in male mice on standard diets or in either males or females maintained on high-fat diets for 8 weeks. Moreover, *Mstn*^*Gdf11/Gdf11*^ and *Mstn*^*+/+*^ mice exhibited similar responses to a glucose challenge in glucose tolerance tests, both in mice maintained on standard diets and in mice maintained on high-fat diets for 4 weeks (Fig. 3C). Hence, complete replacement of MSTN with GDF-11 appeared to have very little effect on glucose metabolism.

In addition to analyzing whether GDF-11 can functionally replace MSTN, we also examined whether increased expression of GDF-11 in these mice affects other biological processes known to be regulated by GDF-11. In particular, we examined whether axial skeleton patterning was affected in *Mstn*^*Gdf11/Gdf11*^ mice. Previous studies have shown that loss of GDF-11 leads to anteriorly directed homeotic transformations of the axial skeleton [28] and that loss of GDF-11 inhibitors, specifically FST and/or GASP-1/GASP-2, leads to posteriorly directed transformations [43, 46]. Analysis of Alizarin red- and Alcian blue-stained skeletons prepared from 25 wild-type and 30 *Mstn*^*Gdf11/Gdf11*^ newborn mice revealed the normal pattern of 7 cervical, 13 thoracic, and 6 lumbar vertebrae in all 55 mice. Moreover, in the cervical region, anterior tuberculi were present on C6 in all mice, and in the thoracic region, the first seven ribs were attached to the sternum in all mice, with the last six ribs remaining floating. Hence, axial skeletal patterning appeared to be normal in *Mstn*^*Gdf11/Gdf11*^ newborn mice and did not exhibit the posteriorly directed homeotic transformations that might be predicted to result from excess GDF-11 activity.

**Fig. 4:**
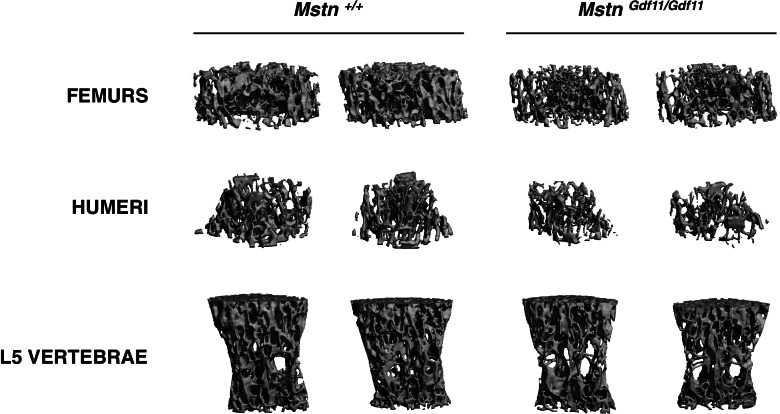
Micro-CT images of representative femurs, humeri, and L5 vertebrae isolated from male *Mstn*^*+/+*^ and *Mstn*^*Gdf11/Gdf11*^ mice

Finally, we examined bones of *Mstn*^*Gdf11/Gdf11*^ mice. *Mstn*^*−/−*^ mice have been reported to have increased bone density [65], possibly as a secondary effect of increased muscling. The role of GDF-11 in regulating bone homeostasis has been controversial, with one study reporting a decrease in bone density upon exogenous administration of GDF-11 to adult mice [66] and another study reporting decreased bone mass not only in newborn *Gdf11* null mice but also in young adult mice in which tamoxifen-inducible cre-mediated recombination was used to target a *Gdf11*^*flox*^ allele [67]. Whatever specific role GDF-11 may play in bone, targeting the MSTN/GDF-11/activin A signaling pathway, either pharmacologically using decoy forms of ACVR2 [68] or ACVR2B [69–73] or genetically by targeting *Acvr2*/*Acvr2b* [74] or *Alk4*/*Alk5* [45] in osteoblasts, has been shown to cause significant increases in bone mass and density. To assess possible effects of the knock-in allele on bone, we first assessed bone density by DXA. As shown in Table 1, we observed a small (~ 5%) but significant decrease in whole body bone density in *Mstn*^*Gdf11/Gdf11*^ male mice compared to *Mstn*^*+/+*^ control mice. Comparable differences were present in multiple regions of the body, including upper and lower limb long bones as well as lumbar vertebrae, but no differences were seen in female mice. To analyze bone structure in greater detail, we carried out micro-CT analysis of humeri, femurs, and lumbar vertebrae. As shown in Table 4 and Fig. 4, numerous micro-CT parameters, including bone surface, BV/TV, connectivity density, trabecular number, trabecular thickness, and bone mineral density, were significantly decreased in *Mstn*^*Gdf11/Gdf11*^ compared to *Mstn*^*+/+*^ male mice; for example, BV/TV and bone mineral density were reduced in femurs by 43% and 48%, respectively, humeri by 26% and 34%, respectively, and L5 vertebrae by 19% and 22%, respectively. Consistent with the DXA data, the only significant differences seen in micro-CT parameters in females were small decreases in BV/TV and bone mineral density (reduced by 8% and 9%, respectively) in L5 vertebrae.

**Table 4:** MicroCT analysis (trabecular bone)

	Male +/*+* (*n* = 9)	Male *Gdf11*/*Gdf11* (*n* = 8)	Female +/*+* (*n* = 7)	Female *Gdf11*/*Gdf11* (*n* = 10)
**Femurs**				
Total volume (mm^3^)	2.16 ± 0.08	1.86 ± 0.05^b^	1.75 ± 0.05	1.72 ± 0.04
Bone volume (mm^3^)	0.403 ± 0.058	0.194 ± 0.015^b^	0.139 ± 0.011	0.121 ± 0.012
Bone surface (mm^2^)	21.49 ± 1.97	13.03 ± 0.87^b^	9.36 ± 0.61	8.43 ± 0.78
Bone volume fraction (BV/TV) (%)	18.20 ± 2.04	10.34 ± 0.65^b^	7.92 ± 0.55	7.01 ± 0.57
Bone surface density (1/mm)	9.82 ± 0.56	6.95 ± 0.33^c^	5.35 ± 0.28	4.86 ± 0.36
Specific bone surface (1/mm)	57.70 ± 3.80	70.85 ± 2.58^b^	71.55 ± 1.77	73.79 ± 1.92
Connectivity density (1/mm^3^)	246.4 ± 12.1	179.1 ± 16.3^b^	104.4 ± 10.5	97.0 ± 13.1
Structure model index	1.81 ± 0.19	2.52 ± 0.07^b^	2.75 ± 0.08	2.81 ± 0.07
Trabecular number (Tb.N) (1/mm)	5.61 ± 0.14	4.75 ± 0.14^c^	4.18 ± 0.08	4.01 ± 0.13
Trabecular thickness (Tb.Th) (μm)	48.0 ± 2.6	40.5 ± 1.8^a^	40.2 ± 0.6	39.5 ± 0.8
Trabecular spacing (Tb.Sp) (μm)	166.5 ± 5.6	204.6 ± 7.0^c^	235.4 ± 4.6	248.3 ± 8.2
Standard deviation of Tb.Th (μm)	17.4 ± 1.5	14.2 ± 1.1	15.0 ± 0.14	15.0 ± 0.68
Standard deviation of Tb.Sp (μm)	53.8 ± 2.5	66.0 ± 2.2^b^	69.3 ± 2.8	75.1 ± 2.9
Degree of anisotropy	1.67 ± 0.05	1.53 ± 0.03^a^	1.61 ± 0.03	1.56 ± 0.04
Bone mineral density (mg/ccm HA)	153.5 ± 18.7	79.7 ± 6.1^b^	54.8 ± 4.9	47.0 ± 5.2
Tissue density (mg/ccm HA)	876.7 ± 7.5	854.5 ± 10.0	856.9 ± 4.6	857.6 ± 4.9
**Humeri**				
Total volume (mm^3^)	0.93 ± 0.02	0.90 ± 0.03	0.82 ± 0.03	0.77 ± 0.02
Bone volume (mm^3^)	0.088 ± 0.007	0.063 ± 0.008^a^	0.054 ± 0.005	0.045 ± 0.005
Bone surface (mm^2^)	5.84 ± 0.44	4.44 ± 0.51^a^	3.47 ± 0.23	2.91 ± 0.29
Bone volume fraction (BV/TV) (%)	9.36 ± 0.59	6.97 ± 0.62^b^	6.52 ± 0.46	5.75 ± 0.52
Bone surface density (1/mm)	6.21 ± 0.37	4.90 ± 0.38^a^	4.21 ± 0.20	3.73 ± 0.32
Specific bone surface (1/mm)	70.86 ± 1.97	76.32 ± 1.84^a^	69.76 ± 2.27	69.73 ± 1.41
Connectivity density (1/mm^3^)	117.5 ± 12.9	80.3 ± 14.4	49.4 ± 5.4	47.0 ± 8.6
Structure model index	2.86 ± 0.06	3.03 ± 0.07	2.89 ± 0.08	3.06 ± 0.12
Trabecular number (Tb.N) (1/mm)	4.99 ± 0.15	4.46 ± 0.13^a^	3.71 ± 0.06	3.63 ± 0.12
Trabecular thickness (Tb.Th) (μm)	40.4 ± 1.2	38.0 ± 1.0	40.6 ± 1.4	41.4 ± 0.9
Trabecular spacing (Tb.Sp) (μm)	195.2 ± 6.8	220.9 ± 6.7^a^	268.7 ± 5.1	277.6 ± 9.5
Standard deviation of Tb.Th (μm)	14.5 ± 0.8	13.5 ± 0.6	15.3 ± 1.0	16.1 ± 0.7
Standard deviation of Tb.Sp (μm)	57.6 ± 1.8	68.5 ± 3.0^b^	94.0 ± 8.0	87.4 ± 5.0
Degree of anisotropy	1.83 ± 0.02	1.80 ± 0.05	1.80 ± 0.04	1.81 ± 0.04
Bone mineral density (mg/ccm HA)	75.5 ± 5.8	50.0 ± 6.1^b^	43.0 ± 3.4	37.1 ± 4.7
Tissue density (mg/ccm HA)	910.4 ± 7.8	901.5 ± 5.8	908.1 ± 7.8	928.5 ± 9.4
**L5 Vertebrae**				
Total volume (mm^3^)	2.06 ± 0.06	1.98 ± 0.05	2.03 ± 0.07	2.04 ± 0.05
Bone volume (mm^3^)	0.541 ± 0.034	0.417 ± 0.020^b^	0.427 ± 0.019	0.397 ± 0.015
Bone surface (mm^2^)	26.63 ± 1.03	22.91 ± 1.04^a^	22.49 ± 0.72	21.41 ± 0.70
Bone volume fraction (BV/TV) (%)	26.05 ± 0.92	20.98 ± 0.56^c^	21.03 ± 0.38	19.40 ± 0.49^a^
Bone surface density (1/mm)	12.90 ± 0.21	11.55 ± 0.28^b^	11.11 ± 0.10	10.48 ± 0.22^a^
Specific bone surface (1/mm)	49.90 ± 1.43	55.45 ± 0.29^b^	53.31 ± 1.03	54.57 ± 0.65
Connectivity density (1/mm^3^)	306.1 ± 10.5	266.9 ± 13.3^a^	280.9 ± 12.0	253.9 ± 7.8
Structure model index	0.46 ± 0.08	0.87 ± 0.05^c^	0.81 ± 0.04	0.91 ± 0.05
Trabecular number (Tb.N) (1/mm)	5.59 ± 0.08	5.21 ± 0.08^b^	4.81 ± 0.06	4.63 ± 0.07
Trabecular thickness (Tb.Th) (μm)	46.3 ± 1.6	41.6 ± 0.2^a^	43.7 ± 0.7	43.1 ± 0.5
Trabecular spacing (Tb.Sp) (μm)	166.2 ± 2.8	180.1 ± 3.5^b^	198.6 ± 3.2	207.4 ± 3.8
Standard deviation of Tb.Th (μm)	13.8 ± 1.0	11.4 ± 0.1^a^	12.6 ± 0.19	12.7 ± 0.27
Standard deviation of Tb.Sp (μm)	73.8 ± 2.2	75.2 ± 4.4	85.2 ± 3.5	91.4 ± 2.8
Degree of anisotropy	2.01 ± 0.04	1.98 ± 0.04	1.96 ± 0.04	1.96 ± 0.02
Bone mineral density (mg/ccm HA)	231.0 ± 8.7	181.3 ± 5.6^c^	180.6 ± 4.1	164.9 ± 4.9^a^
Tissue density (mg/ccm HA)	906.7 ± 6.2	887.9 ± 1.7^a^	873.4 ± 6.6	879.1 ± 3.5

^a^*p* < 0.05 vs. +/+

^b^*p* < 0.01 vs. *+*/+

^c^*p* < 0.001 vs. +/+

Two general conclusions seem evident from the results presented here. First, mature GDF-11 appears to be capable of completely functionally replacing mature MSTN with respect to the control of muscle mass; in fact, based on the slight decreases in muscle mass seen in *Mstn*^*Gdf11/Gdf11*^ male mice, mature GDF-11 made from the knock-in allele actually seems to be even more active than mature MSTN in the control of muscle mass. We did observe trends toward higher levels of circulating GDF-11 resulting from expression of the knock-in allele compared to circulating MSTN levels resulting from expression from the wild-type allele in male mice, but these differences were not statistically significant. This enhanced activity in mice carrying the knock-in allele could reflect either inherent differences in the biological properties of mature MSTN versus mature GDF-11 or differences in levels of activation of the latent MSTN complex versus the hybrid latent complex of mature GDF-11 bound to the MSTN propeptide. Second, complete replacement of mature MSTN with mature GDF-11 does not lead to some of the developmental or physiological changes that might be expected as a result of overexpression of GDF-11. Developmentally, *Mstn*^*Gdf11/Gdf11*^ mice appear to exhibit completely normal axial skeletal patterning. This might not be surprising given that *Gdf11* and *Mstn* have distinct expression patterns during embryogenesis, with *Gdf11* being expressed in the tail bud and primitive streak region in mid-gestation embryos [28] and *Mstn* being expressed in the myotome compartment of developing somites [1]. Despite these distinct expression patterns, however, the two genes are known to be partially functionally redundant with respect to axial patterning, with *Gdf11*/*Mstn* double mutants exhibiting more extensive anteriorly directed homeotic transformations than *Gdf11* single mutants [35]. Hence, the normal skeletal patterning seen in *Mstn*^*Gdf11/Gdf11*^ mice suggests that the biological activities of mature GDF-11 and mature MSTN are similar with respect to regulation of axial patterning. Physiologically, *Mstn*^*Gdf11/Gdf11*^ mice appear to be relatively normal with respect to glucose metabolism despite having circulating GDF-11 levels that are increased ~ 30–40-fold. Based on the report that purified GDF-11 but not MSTN can improve glucose tolerance [57], one might have expected *Mstn*^*Gdf11/Gdf11*^ mice to have improved responses to glucose challenges compared to wild-type mice, but we observed no significant differences between *Mstn*^*Gdf11/Gdf11*^ and wild-type mice in glucose tolerance tests on either standard or high-fat diets. We did observe trends toward lower glucose values in glucose tolerance tests in mice maintained on high-fat diets, but these differences were not statistically significant, and in fact, *Mstn*^*Gdf11/Gdf11*^ female mice actually had slightly elevated fasting glucose levels that were statistically significant.

One tissue that was substantially affected by replacing MSTN with GDF-11 was bone, with *Mstn*^*Gdf11/Gdf11*^ mice exhibiting significantly decreased BV/TV, trabecular number, trabecular thickness, and bone mineral density, at least in males. Although there are conflicting reports as to the role of GDF-11 in regulating bone, the phenotype of *Mstn*^*Gdf11/Gdf11*^ mice is consistent with the effects that have been observed upon blocking this pathway pharmacologically using decoy forms of activin type 2 receptors [68–73] or genetically targeting *Acvr2*/*Acvr2b* [74] or *Alk4*/*Alk5* [45] in osteoblasts. The decreased bone mass seen in *Mstn*^*Gdf11/Gdf11*^ mice would be consistent with the possibility that GDF-11 made from the hybrid precursor protein is slightly more active than endogenous MSTN, as suggested by the slight decreases in muscle mass seen in the knock-in mice. Alternatively, the bone phenotype could result from increased GDF-11 levels per se, perhaps reflecting inherent differences between GDF-11 and MSTN in their ability to regulate bone.

In interpreting the results of these studies, it is important to keep in mind not only that GDF-11 is expressed under the control of *Mstn* regulatory sequences in these knock-in mice but also that GDF-11 protein is made from a hybrid precursor protein containing the MSTN propeptide. Although the MSTN propeptide and GDF-11 propeptide are each capable of binding mature GDF-11 and being cleaved by BMP-1/tolloid proteases to activate latency [37, 38, 40], it is possible that these propeptides have distinct properties that confer some degree of specificity with respect to the biological functions carried out by MSTN versus GDF-11. Hence, additional experiments, such as germline replacement of the MSTN propeptide with the GDF-11 propeptide or the converse germline replacement of the GDF-11 propeptide and/or mature domain with the corresponding portions of MSTN, will be required to understand the full extent to which the various domains of these molecules are functionally equivalent.

Our findings are significant in the context of the current uncertainty surrounding the biological activity of GDF-11 in vivo. A series of papers have suggested that GDF-11 may play a key role in tissue aging. Circulating GDF-11 levels in mice have been reported to decline during aging [75], and injection of purified GDF-11 protein to aged mice was shown to reverse age-related cardiac hypertrophy [75], stimulate vascular remodeling and enhance neurogenesis in the nervous system of aged mice [76], and improve satellite cell function and muscle regeneration and function in aged mice [77]. These studies have suggested that restoring GDF-11 levels to youthful levels may be a new therapeutic strategy to prevent or reverse age-related tissue dysfunction in a wide range of tissues. Some other studies have not found a decline in GDF-11 levels during aging. Subsequent studies have suggested that the assay used to measure circulating levels of GDF-11 may have been detecting circulating MSTN and that GDF-11 levels are either unchanged or perhaps even increased during aging [59, 78, 79]. The reported effect of GDF-11 in promoting muscle regeneration also has remained uncertain; this finding was unexpected given that loss or inhibition of MSTN signaling had been shown to improve muscle regeneration in the setting of both acute muscle injury and chronic muscle degeneration (for reviews, see references [80–82]). Indeed, subsequent studies reported that administering purified GDF-11 to mice impairs the ability of the muscle to regenerate [78, 83], which is more consistent with the fact that MSTN and GDF-11 are virtually indistinguishable in their biological properties in vitro. Although it is possible that differences in the experimental procedures and dose regimens used may account for the discrepant findings among studies and that exogenously administered GDF-11 may behave differently than endogenously produced MSTN, our findings are consistent with GDF-11 being capable of functionally replacing MSTN in vivo.

## Data Availability

All data generated or analyzed during this study are included in this published article.

## Abbreviations

MSTN: Myostatin
TGF-ß: Transforming growth factor-ß
ES: Embryonic stem
DXA: Dual-energy X-ray absorptiometer
GTT: Glucose tolerance test
HU: Hounsfield unit
